# Multi- analytical approach: better predictor of pharmacogenetic based clinical outcomes in breast cancer therapies

**DOI:** 10.1186/1755-8166-7-S1-P99

**Published:** 2014-01-21

**Authors:** Sonam Tulsyan

**Affiliations:** 1 Department of Genetics, Sanjay Gandhi Post Graduate Institute of Medical Sciences, Lucknow, India

## Background

Chemotherapeutic drug clinical outcomes are genetically determined as there is a large heterogeneity in the response to, and toxicity of, chemotherapeutic agents in breast cancer patients. Polymorphisms in genes encoding Phase I – Cytochrome P450 (CYP450) and NAD(P)H dehydrogenase quinine (NQO1); phase II - glutathione-Stransferase (GST) and methylene tetra hydrofolate reductase (MTHFR); phase III- p-glycoprotein (ABCB1) and solute transporter (SLC22A16) drug metabolizing enzymes can possibly predict clinical outcomes, and can be of prognostic significance in breast cancer patients. The aim of this study was to determine the role of genetic variations in drug metabolizing enzymes in predicting response and toxicity in breast cancer patients, using multi-analytical approaches.

## Materials & methods

Two hundred and four North Indian cases of histology proven invasive breast carcinoma treated at the our institute were genotyped for 17 polymorphisms by Polymerase Chain Reaction (PCR) or PCR-Restriction Fragment Length Polymorphism (RFLP) or Taqman allelic discrimination assay. All patients were treated with combination chemotherapy containing an anthracycline drug—epirubicin (62.70 %) or doxorubicin (37.25%) along with cyclophosphamide and 5-fluorouracil. Tumor response to NACT was recorded in 96 patients according to Response Evaluation Criteria in Solid Tumors (RECIST). Toxicological data was recorded according to National Cancer Institute- Common Terminology Criteria for Adverse Events (NCI-CTCAE). The highest grade toxicity that occurred in an individual patient during the course of treatment was used for the analysis. Genetic variations were correlated with response to NACT and chemo-toxicity using logistic regression through SPSS software, version 17.0. Furthermore, higher-order gene-gene interactions were determined through multifactor dimensionality reduction (MDR) using software version 2.0 beta8.

## Results

Heterozygous (CT) and variant (TT) genotype of ABCB1 1236C>T polymorphism was found to be significant with breast cancer response to NACT. Similarly, heterozygous (CT) genotype of ABCB1 1236C>T polymorphism was significantly associated with grade 2-4 anemia. Furthermore, we found significant association of heterozygous genotype (*1/*3) of CYP2C9*3 with grade 2-4 leucopenia and CT genotype of NQO1 polymorphism with dose delay/ reduction. However, none of the polymorphisms were found to be statistically significant with grade 2-4 toxicity. On MDR analysis, ABCB1 1236C>T polymorphism yielded the highest testing accuracy for response to NACT (CVT=0.61, CVC= 10/10, p =0.0067). However, ABCB1 1236C>T, ABCB1 2677G>T/A, CYP2C19*2 combination of polymorphisms yielded the highest testing accuracy for grade 2-4 anemia (CVT=0.66, CVC= 10/10, p < 0.0001). Similarly, the above combination of polymorphisms provided the best interaction model for overall toxicity as well (CVT=0.65, CVC= 10/10, p < 0.0001). For dose delay/ reduction, NQO1 609 C>T polymorphism yielded the highest testing accuracy (CVT=0.60, CVC= 9/10, p =0.0048). However, CYP2B6*9 polymorphism with the testing accuracy (CVT=0.43, CVC= 5/10) for grade 2-4 leucopenia did not achieve statistical significance (p= 0.1088).

## Conclusions

Multi-analytical approaches may provide a better prediction of pharmacogenetic based clinical outcomes in breast cancer patients.

